# A Review of Transition Metal Phosphides for Hydrazine-Assisted Electrolytic Water Splitting for Hydrogen Production

**DOI:** 10.3390/nano16140874

**Published:** 2026-07-16

**Authors:** Minghao Yuan, Jun Wang, Xiaoqing Liao, Junhan Wang, Minghao Bian, Jingwen Ma

**Affiliations:** 1 School of Chemical and Environmental Engineering, China University of Mining and Technology (Beijing), Beijing 100083, China; zqt2500303183@student.cumtb.edu.cn (M.Y.); 2410320105@student.cumtb.edu.cn (X.L.); 2410320325@student.cumtb.edu.cn (J.W.); 2410320211@student.cumtb.edu.cn (M.B.); 2 PetroChina Planning and Engineering Institute, Beijing 100083, China

**Keywords:** transition metal phosphides, hydrazine oxidation reaction, energy-efficient hydrogen production, design strategies, applications

## Abstract

Electrochemical water splitting for hydrogen production is an important path for the preparation of green hydrogen. However, the sluggish kinetics and high energy consumption of the anode oxygen evolution reaction (OER) have restricted its development. The hydrazine oxidation reaction (HzOR), with its low theoretical potential, fast kinetics, clean products, and the ability to simultaneously treat hydrazine-containing wastewater, has emerged as an ideal anode reaction to replace OER. Transition metal phosphides (TMPs) have shown noble-metal-like activity in HzOR catalysis due to their tunable d-band electronic structure, abundant active sites, high conductivity, and structural stability, making them highly promising non-noble metal catalysts. However, most existing reviews focus on the catalytic performance of TMPs in general hydrogen evolution reaction (HER)/OER systems or merely briefly mention HzOR as one of many anode reactions. Therefore, this review aims to comprehensively and systematically elaborate on the design strategies of TMPs catalysts for hydrazine-assisted electrolytic water splitting for hydrogen production and their applications in HzOR, deeply discuss the current progress, challenges, and future directions, and provide references for the development and industrial application of low-cost, high-efficiency, and high-stability hydrazine-assisted hydrogen production catalysts.

## 1. Introduction

Driven by the global energy transition and the dual carbon goals, the development of efficient and clean energy conversion technologies has become an urgent priority [1,2]. Hydrogen, due to its high energy density (142 MJ/kg) and zero-carbon emission characteristics, is regarded as the core carrier of future energy systems [3]. Compared to other hydrogen production processes, electrochemical water splitting technology can be driven by renewable energy to achieve truly green hydrogen production, and has therefore attracted significant attention [4,5,6]. However, traditional water electrolysis systems are limited by the thermodynamic bottleneck of the anode oxygen evolution reaction (OER). OER involves a complex four-electron transfer process ($2H_{2}O\to O_{2}+4H^{+}+4e^{-}$), with a theoretical potential as high as 1.23 V vs. RHE. Due to its slow kinetics, an additional overpotential of 0.3–0.5 V or even higher must be applied, resulting in persistently high overall energy consumption [7]. This high energy consumption severely limits the economic competitiveness of water electrolysis technology. Consequently, researchers are actively exploring thermodynamically more favorable alternative anode reactions, among which small-molecule oxidation reactions have garnered significant attention due to their advantages of low theoretical potential, fast reaction kinetics, and high product value [8,9,10,11].

Hydrazine (N_2_H_4_) is a nitrogen-containing compound with a high energy density (19.5 MJ/kg) that has a long history of application in fields such as space propulsion, chemical synthesis, and fuel cells. The theoretical potential of its electrochemical oxidation reaction (HzOR) is only −0.33 V vs. RHE, which reduces the theoretical cell voltage by approximately 1.56 V compared to the oxygen evolution reaction (OER, 1.23 V vs. RHE) [12,13]. Furthermore, the products of HzOR are N_2_ and H_2_O, resulting in no greenhouse gas emissions; nitrogen can be directly released into the atmosphere without post-treatment, combining energy production with environmental benefits [14,15,16,17]. However, hydrazine itself is a highly toxic and carcinogenic substance, and its storage, transportation, and usage pose significant safety risks, which constitute a major constraint on the large-scale application of this technology [18,19]. Therefore, using HzOR instead of OER can realize energy-saving hydrogen production, meanwhile degrading hydrazine in wastewater. In 2017, Sun et al. [20] pioneered groundbreaking research on replacing OER with HzOR as the anode reaction. The team prepared nickel phosphide (Ni_2_P) nanoarrays (Ni_2_P/NF) supported on nickel foam and utilized them as a bifunctional electrocatalyst for both HzOR and the hydrogen evolution reaction (HER), achieving highly efficient and energy-saving hydrogen production. In a 1.0 M KOH solution containing 0.5 M hydrazine, this hydrazine-assisted electrolysis system required only 1.0 V to drive a high current density of 500 mA·cm^2^, reducing energy consumption by more than 80% compared to conventional open water system (OWS) electrolyzers, thereby opening up new avenues for the development of low energy consumption electrochemical hydrogen production technologies.

**Figure 1:**
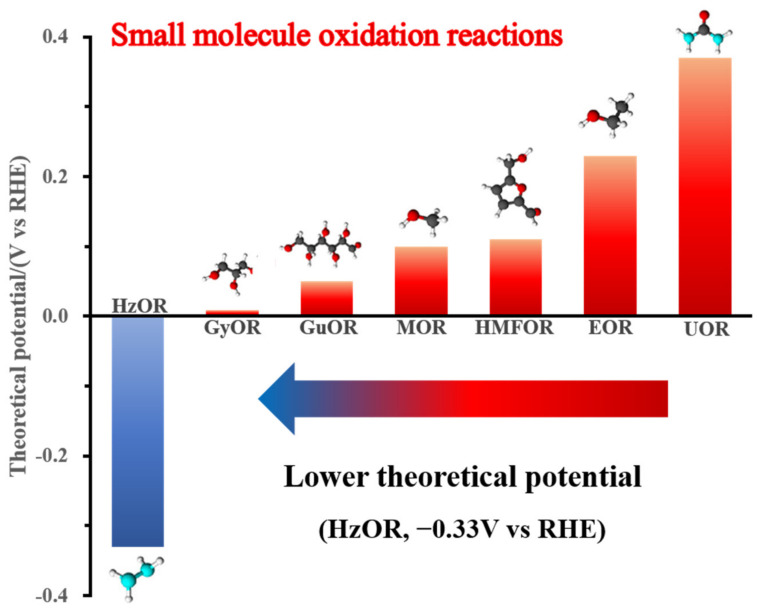
Comparison of HzOR with other oxidation reactions.

Compared with other alternative anode reactions, HzOR demonstrates unique advantages (Figure 1). Although the urea oxidation reaction (UOR) also features a low theoretical potential (0.37 V) and environmental benefits, it involves a six-electron transfer process, exhibits slow reaction kinetics, and the complex molecular structure of urea leads to cumbersome intermediate adsorption/desorption steps [21]. The alcohol oxidation reaction (AOR), on the other hand, faces challenges in controlling product selectivity, resulting in high costs for separation and purification [22,23]. In contrast, the 4-electron transfer pathway in HzOR is simple and well-defined. N_2_ is generated directly after N-N bond cleavage, with selectivity approaching 100%, and the reaction kinetics are significantly superior to those of UOR and AOR [24,25,26].

Due to various factors, the reaction pathways differ under different reaction conditions and catalyst systems, leading to significant variations in the reaction mechanisms of the HzOR electrocatalyst. Compared to acidic and neutral media, the kinetics of HzOR are optimal in alkaline media (e.g., 1 M KOH), where high concentrations of OH^−^ promote the initial deprotonation of N_2_H_4_ to form the N_2_H_3_^−^ intermediate, significantly lowering the reaction energy barrier [27]. In acidic media (e.g., 0.5 M H_2_SO_4_), HzOR follows the $N_{2}H_{4}\to N_{2}+4H^{+}+4e^{-}$ pathway. While a high proton concentration environment favors the synergistic dehydrogenation process, precious metal catalysts (such as Pt) are prone to poisoning due to the strong adsorption of nitrogen-containing intermediates, limiting their practical application [28]. Under neutral conditions (e.g., phosphate buffer), water molecules serve as the primary medium for proton transfer. The reaction pathway involves a coupled process of deprotonation and electron transfer, and the kinetics are significantly influenced by the buffering capacity [29]. Therefore, current research primarily focuses on HzOR under basic conditions, as the reaction pathway is relatively well-defined, the catalytic performance is superior, and it holds greater potential for practical applications. In alkaline electrolyte solutions, the electrode reactions for hydrazine electrolysis are as follows: Under alkaline conditions, HER involves either a two-electron transfer reaction following the Volmer–Tafel (V–T) mechanism or a four-electron transfer reaction through four consecutive dehydrogenation processes. The corresponding reaction processes can be described by the Volmer–Heyrovsky (V–H) reaction mechanism, and HzOR is typically described using Equations (1)–(8) [30,31].

(1) $$\begin{array}{c} \mathrm{Cathodic}\;\left(HER\right):\;4H_{2}O+4e^{-}\to 2H_{2}+4{OH}^{-} \end{array}$$

(2) $$\begin{array}{c} Volmer:\;H_{2}O+e^{-}\to H^{*}+{OH}^{-} \end{array}$$

(3) $$\begin{array}{c} Tafel:\;H^{*}+H^{*}\to H_{2} \end{array}$$

(4) $$\begin{array}{c} or\;Heyrovsky:\;H^{*}+H_{2}O+e^{-}\to H_{2}+{OH}^{-} \end{array}$$

(5) $$\begin{array}{c} Anodic\;\left(HzOR\right):\;N_{2}H_{4}+4{OH}^{-}\to N_{2}+4H_{2}O+4e^{-} \end{array}$$

(6) $$\begin{array}{c} N_{2}H_{4}+{OH}^{-}\to {N_{2}H_{3}}^{*}+H_{2}O+e^{-} \end{array}$$

(7) $$\begin{array}{c} {N_{2}H_{3}}^{*}+{OH}^{-}\to {N_{2}H_{2}}^{*}+H_{2}O+e^{-} \end{array}$$

(8) $$\begin{array}{c} {N_{2}H_{2}}^{*}+{OH}^{-}\to N_{2}H^{*}+H_{2}O+e^{-} \end{array}$$

(9) $$\begin{array}{c} N_{2}H^{*}+{OH}^{-}\to N_{2}+H_{2}O+e^{-} \end{array}$$

In summary, the development of highly efficient, stable, and low-cost HzOR electrocatalytic materials has become a key research focus. Transition metal phosphides (TMPs), with their tunable d-band electronic structure and abundance of active sites, are highly promising non-precious metal catalysts. Following modification and optimization through doping and heterojunction formation, some TMPs exhibit catalytic performance comparable to commercial Pt/C in alkaline laboratory electrolytes, and their overall stability generally surpasses that of pure nickel and cobalt [32,33]. In particular, the recently reported in situ regeneration mechanism of active sites in NiCoP bimetallic phosphides has effectively mitigated structural degradation of the catalyst under high current densities, achieving industrial-scale current densities as high as 1000 mA·cm^−2^ and long-term operational stability exceeding 100 h. This indicates that TMP-based catalysts hold considerable potential for industrial development in the field of hydrazine oxidation electrolysis [34]. However, most existing reviews have focused on the catalytic performance of TMPs in HER/OER systems, or have only briefly mentioned HzOR as one of many anodic reactions. Therefore, this review focuses on the field of hydrogen production via hydrazine-assisted electrochemical oxidation and presents the latest advances in the design strategies and applications of TMPs as electrocatalysts. Specifically, Section 2 systematically discusses the catalytic advantages of TMPs and their three main preparation methods: liquid-phase synthesis, gas–solid reactions, and electrodeposition, laying the theoretical foundation for the subsequent discussion of design strategies. Section 3 examines in detail the roles of morphological engineering, metal doping, heterostructure construction, and other auxiliary regulation strategies (such as local restructuring and ligand modulation) in optimizing the electronic structure and reaction pathways of TMPs. Section 4 highlights the latest advances in TMP-based catalysts across application areas such as direct hydrazine fuel cells, hydrazine-assisted seawater electrolysis for hydrogen production, and the purification of industrial hydrazine-containing wastewater, demonstrating their potential for synergistic energy and environmental management. Finally, Section 5 discusses the key challenges currently faced in the transition from the laboratory to industrialization (such as green synthesis, the dynamic evolution of active sites, performance retention under low-concentration hydrazine conditions, and membrane–electrode integration), as well as emerging opportunities and future research directions. This review aims to provide valuable references and insights for researchers dedicated to developing low-cost, highly efficient, and highly stable transition metal phosphide-based catalysts for hydrazine-assisted water electrolysis for hydrogen production.

## 2. Transition Metal Phosphides

### 2.1. Catalytic Advantages

Due to their unique electronic structure and catalytic properties, TMPs have attracted widespread attention in the field of hydrogen production via electrolytic oxidation of hydrazine with a co-catalyst [35,36]. Compared with sulfides (such as MoS_2_), the active sites of TMPs are not limited to the edges. Both the bulk and the surface can provide abundant catalytic centers [37]. Metal oxides and hydroxides, on the other hand, face the issue of high charge transfer resistance, which severely hinders the electron transfer process and consequently inhibits the kinetics of catalytic reactions [38,39]. Although metal carbides and nitrides exhibit high activity in HER and OER, their active site density in HzOR is typically lower than that of phosphides, and their synthesis conditions are harsh. These processes often involve high-temperature treatment or annealing steps following ammonia reduction, both of which not only increase production costs but also exacerbate environmental burdens [40,41].

In contrast, TMPs exhibit a precious-metal-like electronic structure and a tunable d-band center. Phosphorus atoms have high electronegativity (2.19). When bonded to transition metals (such as Ni, Co, and Fe), they induce delocalization of the metal d-orbitals, thereby optimizing the adsorption energy of the metal center for N_2_H_4_ molecules and the activation energy barrier of the N–H bond. Density functional theory (DFT) studies indicate that the d-band centers of phosphides such as Ni_2_P and CoP are positioned close to those of platinum (Pt). This gives them intrinsic electronic properties similar to noble metals, enabling them to absorb N_2_H_4_ molecules efficiently and lower the activation energy barrier for N–H bond cleavage. This is a prerequisite for the HzOR reaction to progress rapidly [42]. More importantly, the position of the d-band center dictates the adsorption affinity of reaction intermediates. An overly low d-band center leads to insufficient adsorption, which hinders the activation of reactants, whereas an overly high d-band center causes excessively strong binding, retarding product desorption. For example, by adjusting the d-band center of Ni_2_P through Mn doping, the energy barrier for the dehydrogenation step of N_2_H_4_ can be reduced from 1.09 eV to 1.02 eV. At the same time, the hydrogen adsorption free energy can be optimized from −0.32 eV to −0.08 eV, approaching thermodynamic neutrality. Thus, the kinetics of HzOR and HER can be simultaneously enhanced [43].

At the same time, TMPs possess a unique bifunctional synergistic catalytic mechanism. In the HzOR process, the transition metal sites are primarily responsible for N–H bond cleavage, while the adjacent phosphorus atoms play a crucial auxiliary role. The phosphorus sites not only stabilize the dehydrogenated intermediates (e.g., N_2_H_3_*), but also promote the adsorption of OH^–^ ions in the electrolyte, thereby accelerating subsequent deprotonation or coupling steps (e.g., $2N_{2}H_{3}\to N_{2}+2{NH}_{3}$). Specifically, the lone pair electrons of the phosphorus atom can form feedback bonds with the d orbitals of the metal, regulating the electron density at the metal site and making it more favorable for the end group adsorption of N_2_H_4_. At the same time, the phosphorus site itself can act as a proton acceptor, assisting in the transfer of H, thereby reducing the apparent activation energy of the entire multi-step dehydrogenation process. This metal-phosphorus synergy is absent in single-component metal oxides or sulfides, which typically provide only a single type of active site and struggle to efficiently drive multistep reactions [44,45]. Coupled with the high strength and toughness of metal-phosphorus bonds (e.g., the Ni–P bond energy is approximately 370 kJ/mol), although TMPs undergo surface restructuring at anodic potentials (with phosphorus partially leaching out and converting to metal hydroxyl oxides), this dynamic evolution works synergistically with the stable bulk structure. This synergy facilitates the exposure more active sites and suppresses excessive metal leaching. As a result, TMPs exhibit excellent stability in harsh electrochemical environments, with an activity decay of less than 5% after a 100 h constant-current test [46,47]. Additionally, its intrinsic electrical conductivity (10^3^–10^4^ S/m) is far higher than that of corresponding metal oxides (10^−6^–10^−2^ S/m), significantly enhancing charge transfer kinetics and reducing ohmic losses, thereby meeting the requirements for high-current-density, efficient hydrogen production. In alkaline media, the phosphorus atoms on the TMPs surface can form a PO_4_^3−^/HPO_4_^2−^ buffer layer [48,49], effectively suppressing side reactions such as the OER and increasing the selectivity of HzOR to over 95%, far surpassing traditional catalysts such as NiO (Faraday efficiency below 80%). This directly reduces system energy consumption and enhances hydrogen production efficiency. Furthermore, the synthesis strategies for TMPs are flexible and easily scalable. The nano-morphology of metal precursors such as MOFs, hydroxides, and oxides can be precisely controlled by phosphating. This can produce hollow nanorods, core–shell structures, and ultrathin nanosheets. This approach not only maximizes the exposure of active sites but also enriches unsaturated coordination atoms through the exposure of high-index crystal faces (e.g., CoP(111)), further enhancing intrinsic activity [50,51,52]. For example, researchers achieved highly efficient HzOR performance at industrial-scale current densities by constructing Fe-NiCoZnP/MXene heterojunctions [53]. Meanwhile, doped CoP catalysts significantly accelerated HzOR-coupled HER by regulating adsorption sites [54]. In recent years, the design of transition metal phosphide compositions has expanded to include high-entropy metal phosphides, which integrate five or more metal elements into a single phosphide lattice. Entropy-stabilized multimetallic configurations not only enhance the material’s structural tolerance against elemental segregation but also form adsorption sites with continuously distributed d-band centers, thereby optimizing the adsorption/desorption equilibrium of various reaction intermediates [55,56]. Regarding HzOR, Li et al. [57] prepared a P-modified amorphous high-entropy CoFeNiCrMn compound (CoFeNiCrMnP/NF) on foam nickel via a one-step electrodeposition method. This catalyst exhibited a HzOR overpotential of 268 mV at a current density of 100 mA·cm^−2^. The assembled hydrazine-assisted electrolyzer achieved a cell voltage of only 91 mV at 100 mA·cm^−2^, which is approximately 1.54 V lower than that of conventional HER||OER systems. The study indicated that the introduction of P and the Cr element significantly contributed to the improvement in HzOR performance.

These properties enable TMPs to exhibit excellent catalytic performance in HzOR. Representative TMP systems include Ni_2_P, CoP, FeP, Cu_3_P, and their bimetallic derivatives (NiCoP, NiCuP, Fe-CoP, etc.). Among these, Ni-based phosphides show superior performance due to their moderate binding energy with the HzOR intermediate. Recent studies have shown that strategies such as morphological engineering (nanosheets, nanowire arrays), metal doping (Cu, Mn), and heterostructure construction (phosphide/phosphide, phosphide/precious metal) can further enhance the HzOR activity, selectivity, and stability of TMPs [58,59].

### 2.2. Synthesis Method

#### 2.2.1. Liquid-Phase Synthesis Method

The liquid-phase synthesis strategies for TMPs primarily involve thermal decomposition of organometallic precursors and anion exchange methods, enabling precise control over the crystalline phase, size, and morphology of their nanostructures. The thermal decomposition method typically utilizes organometallic salts (such as acetylacetonate metal salts) reacting with trioctylphosphine (TOP) in high-boiling-point organic solvents. By precisely controlling the ratio of phosphine/metal precursor, reaction temperature and surface surfactant, a controllable synthesis of various single-phase nanocrystals with different structures (from hollow to solid, from Ni_12_P_5_ to Ni_5_P_4_) was achieved [60]. This formation process is often accompanied by nanoscale Kirkendall effects. Conversely, the anion exchange method employs inorganic -organic hybrid materials (e.g., Fe_18_S_25_-triethylenetetramine nanosheets) as templates. By taking advantage of the dual effects of TOP at high temperatures, that is, acting simultaneously as a phosphorus source and an extraction agent for template anions (such as S^2−^), the template was successfully converted into nano-phosphides with macroscopic morphology (such as FeP). This strategy demonstrates potential for extension to other phosphide systems [61].

However, these advanced liquid-phase synthesis routes, while demonstrating exceptional control capabilities, also face multiple significant challenges. First, from a practical application perspective, these methods generally rely on expensive, air-sensitive, and toxic organic reagents (such as TOP and oleylamine) and are conducted under high-temperature inert atmospheres. Furthermore, while liquid-phase synthesis strategies offer unique advantages in nanoscale control, significant technical challenges remain regarding how to achieve uniform loading and consistent dispersion of nanoparticles on large-area substrates when the electrode area is scaled up from the centimeter to the decimeter or even square meter scale. The nucleation kinetics, mass transfer gradients, and temperature field distribution in solution all exhibit significant scaling effects in large-scale systems, leading to variations in catalyst loading, particle size, and composition across different regions of the substrate, which in turn affects the consistency of the overall electrode’s electrochemical performance [62,63]. Second, the pursuit of precise process control remains constrained by the intricate reaction mechanisms involved. Whether examining the formation of metal nanoclusters in thermal decomposition, the diffusion and phase-transition kinetics of phosphorus atoms, or the equilibrium between ion exchange and template structural evolution in anion exchange, the microscopic details remain unclear. This ambiguity leads to significant uncertainty in reproducibly controlling product morphology, size, and structure, often necessitating empirical exploration. Finally, organic surfactants introduced to stabilize nanostructures form dense coating layers post-synthesis, severely shielding catalytic active sites. The high-temperature annealing treatment carried out to remove the ligands is prone to cause particle sintering, agglomeration or unexpected phase transformation, resulting in a decrease in specific surface area and performance degradation. This constitutes a pressing contradiction that needs to be resolved. Therefore, a key future direction for liquid-phase synthesis research lies in developing greener, more cost-effective precursor routes and advancing mild yet efficient surface treatment techniques to fully expose and stabilize active sites.

#### 2.2.2. Gas–Solid Reaction Method

The gas–solid reaction synthesis of transition metal phosphides typically employs sodium hypophosphite (NaH_2_PO_2_) as the phosphorus source. During thermal treatment, it decomposes to generate PH_3_ gas, which reacts with metals or their precursors (such as oxides, sulfides, or hydroxides) to form corresponding metal phosphides [64]. This method offers advantages including operational simplicity, low cost, applicability to diverse substrates (e.g., nickel foam, cobalt foam, carbon cloth), and morphology control (e.g., nanosheets, nanorods, nanoblossoms). It enables the construction of self-supporting electrodes, facilitating electron conduction and mass transfer to enhance electrocatalytic performance [65,66]. However, the release of toxic and corrosive PH_3_ during the reaction process imposes stringent requirements on experimental safety and control. Precise control of phosphorization temperature and duration is critical for determining crystal structure, porosity, and active site exposure. Temperatures that are too high or too low may result in structural damage or incomplete phosphorization. In addition, the gas–solid reaction method offers certain advantages for the preparation of large-area electrodes, as it does not require the use of solvents and allows catalysts to be grown directly on self-supporting substrates. However, when the reactor is scaled up, significant challenges arise regarding the uniform deposition of solid precursors, including the uniform spatial distribution of PH_3_ gas and the equilibrium of the temperature field within the reactor. Temperature gradients between regions close to and far from the heat source can lead to variations in the degree of phosphorization, which in turn affects the uniformity of catalytic activity across different areas of the large-area electrode [67].

#### 2.2.3. Electrodeposition Method

Electrodeposition of transition metal phosphides offers an effective method for preparing self-supporting phosphide electrodes directly on conductive substrates at ambient temperature and pressure. This method typically employs an electrolyte containing metal salts and sodium hypophosphite. Through constant-current or constant-potential deposition on three-dimensional structures such as foam nickel or carbon nanotube-modified substrates, it forms metal phosphide films or nanoparticles with amorphous/crystalline composite characteristics in a single step [68]. Electrodeposition offers distinct advantages, including simple process steps, low operating temperatures, no need for binders, controllable microstructure and composition (e.g., Co/CoP, NiCoP), and strong adhesion between catalyst and substrate [69]. These features enhance electron transport efficiency and catalytic stability, particularly demonstrating low overpotentials and excellent long-term durability in alkaline hydrogen evolution reactions. However, precise control over phosphorus incorporation and stoichiometric ratios during deposition is challenging, often leading to non-targeted phases or compositional inhomogeneities. Additionally, critical properties like film thickness, porosity, and active site density exhibit extreme sensitivity to process parameters, complicating optimization. Deposition at high current densities may induce stress accumulation or structural densification, hindering electrolyte permeation and gas diffusion. Furthermore, the preparation of highly dispersed, submicron-sized, and uniformly distributed nanostructures remains challenging. In particular, as the electrode area increases, the nonuniformity of current density and electric field distribution becomes significantly more pronounced, leading to variations in the composition, morphology, and thickness of the catalyst across the substrate. The distance between the working electrode and the counter electrode directly affects ion transport kinetics and deposition uniformity. While the distance between the working electrode and the reference electrode significantly influences the precision of controlling the actual deposition potential. these parameters must all be systematically re-optimized during the scaling-up process [70]. Therefore, despite demonstrating unique advantages in integrated electrode construction, electrodeposition still has room for improvement in precisely regulating the intrinsic structure of materials and scaling up to large-scale production.

## 3. Design Strategies for High-Efficiency HzOR Catalysts

HzOR is the core reaction in hydrazine-assisted hydrogen production, and the activity, selectivity and stability of the catalyst directly determine the energy efficiency of the system. To address critical issues in traditional catalytic materials, such as insufficient active sites, slow electron transport and unreasonable intermediate adsorption energies, precise design must be conducted at multi-scale structural and electronic levels. Consequently, establishing a systematic and precise catalytic regulation strategy based on factors such as material morphology, electronic configuration, interfacial properties and surface microenvironment is key to overcoming the HzOR catalytic bottleneck. Based on this, this paper elaborates systematically on the core design concepts and mechanisms of highly efficient HzOR electrocatalysts from four perspectives: morphological engineering, metal doping modification, heterostructure construction and other auxiliary control methods. This provides a theoretical basis and technical reference for developing and applying high-performance catalysts for hydrazine oxidation hydrogen production.

### 3.1. Morphology Engineering

Nanostructure design is a key strategy for enhancing the performance of electrocatalytic materials. Its core objective is to achieve optimization across three levels through precise morphological control: maximizing specific surface area to provide more reaction interfaces, selectively exposing highly active crystal faces to optimize intrinsic catalytic activity, and constructing efficient mass transfer networks to facilitate rapid transport of reactants and products. For the HzOR system, rational morphological design directly influences the accessibility of active sites, the adsorption/desorption kinetics of reaction intermediates, and the long-term stability of the catalyst.

**Figure 2:**
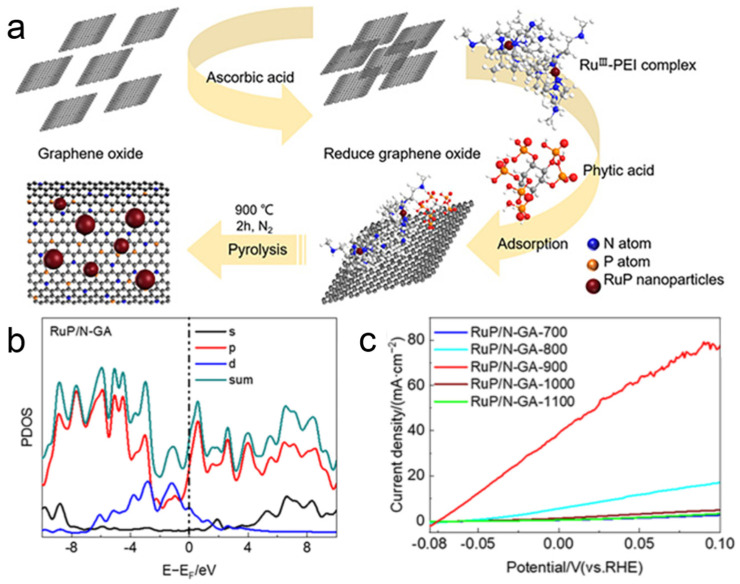
(**a**) Overall synthetic route of RuP/N-GA-900 hybrids. (**b**) PDOS of RuP/N-GA, (**c**) LSV curves of RuP/N-GA-700, RuP/N-GA-800, RuP/N-GA-900, RuP/N-GA-1000 and RuP/N-GA-1100 in 1 mol∙L^−1^ KOH + 0.4 mol∙L^−1^ N_2_H_4_ electrolyte. Reproduced with permission from ref. [71]. Copyright 2023 Acta Physico-Chimica Sinica.

Taking the nanosheet structure as an example, its two-dimensional, ultra-thin configuration maximizes the exposure of surface atoms, significantly increasing the number of active sites available for hydrazine molecule adsorption and activation, thereby enhancing catalytic efficiency. Ni-Co-Fe-P amorphous nanosheets prepared by Darband et al. [47] via an electro-deposition method achieved a current density of 10 mA·cm^−2^ at just 25 mV vs. RHE in an alkaline HzOR, outperforming their OER performance (which required 1.490 V vs. RHE to reach the same current density). The excellent HzOR catalytic activity of this electrode can be attributed to the large specific surface area and abundant exposed sites resulting from the ultrathin nanosheet structure, as well as the synergistic electronic regulation between P and the multi-metal components Ni, Co, and Fe, both of which collectively enhance the material’s intrinsic catalytic activity. DFT analysis indicates that the d-band center of Ni-Co-Fe-P (−1.43 eV) is farther from the Fermi level than that of Ni-Co-P (−1.37 eV) and Ni-Fe-P (−1.35 eV). Due to the proximity of the d-band center to the Fermi level, binary phosphides exhibit excessively strong adsorption of nitrogen-containing intermediates, which can easily lead to site passivation and restricted reaction kinetics. In contrast, the moderately shifted d-band center in the ternary system weakens this excessive adsorption, balances the adsorption of intermediates with the desorption of products, optimizes the stepwise dehydrogenation reaction pathway of hydrazine, and further enhances the overall HzOR catalytic efficiency. Similarly, Wang et al. [71] prepared a bifunctional RuP/N-GA catalyst consisting of ultra-small RuP nanoparticles anchored on nitrogen-doped graphene aerogel (N-GA) via an adsorption–phosphidation method, with RuP/N-GA-900 exhibiting excellent catalytic performance in the HzOR, achieving a current density of 10 mA·cm^−2^ at a low potential of −54 mV vs. RHE (Figure 2c). N-GA exhibits a three-dimensional porous sheet-like framework with a BET specific surface area as high as 200.4 m^2^ g^−1^ and a pore volume of 611.9 cm^3^ g^−1^, providing stable anchoring sites for RuP nanoparticles, preventing their agglomeration, and accelerating electron transport and mass transfer processes (Figure 2a). The supported RuP nanoparticles are all smaller than 5 nm, exhibit a sub-nanometer size distribution, and are uniformly dispersed on the GA surface, exposing abundant active sites. Simultaneously, the low-crystallinity structural characteristics further enhance the accessibility of these active sites. Furthermore, when this catalyst was used in a hydrogen decomposition (O_2_/H_2_) electrolyzer assembled as a bifunctional electrode, it achieved highly efficient hydrogen production at a cell potential of only 41 mV under a current density of 10 mA·cm^−2^, which is significantly lower than the 202 mV required by commercial Pt/C electrolyzers. Such high activity can be attributed to the structural advantages of RuP/N-GA-900: (1) the ultra-small RuP nanoparticles provide abundant Ru active sites; (2) the synergistic effect between N doping in the GA framework and Ru phosphidation enhances electrocatalytic activity; and (3) the 3D porous N-GA with a few-layer structure facilitates electron and mass transport. Furthermore, as clearly shown by the total and partial density of states (PDOS) results (Figure 2b), the electronic distribution of RuP/N-GA-900 is continuous near the Fermi level, indicating that it exhibits metallic behavior and demonstrates excellent electrical conductivity in electrocatalysis.

For one-dimensional nanowire arrays, the rapid axial electron transport and abundant radial open channels provide structural advantages for electrocatalytic reactions. More importantly, the three-dimensional interwoven structure can induce localized electric field concentration, which further modulates the surface charge density and the position of the d-band center. Zhang et al. [72] fabricated a heterogeneous nanowire array catalyst (N-Ni_5_P_4_@CoP/CFP) composed of CoP and N-doped Ni_5_P_4_ on carbon fiber paper using a hydrothermal–phosphidation coupling strategy. In a 1.0 M KOH + 0.1 M N_2_H_4_ electrolyte, a current density of 10 mA·cm^−2^ was achieved at a low potential of only −32 mV vs. RHE, outperforming N-Ni_5_P_4_/CFP (50 mV), CoP/CFP (−21 mV), and commercial Pt/C/CFP (154 mV). The nanowire arrays vertically grown on carbon fiber paper exhibit a distinct interdigitated structure with surfaces rich in dendritic, multi-level pore channels.with a BET specific surface area of 31.77 m^2^ g^−1^ and an average pore diameter of 7 nm. This not only provides abundant exposed active sites but also endows the catalyst with superhydrophilicity (water contact angle close to 0°) and superhydrophobicity (bubble contact angle of 142°), accelerating electrolyte wetting and bubble desorption, thereby enhancing mass transfer efficiency. DFT calculations indicate that the d-band center of this heterojunction is −2.13 eV, which is closer to the Fermi level than that of CoP (−2.35 eV) and N-Ni_5_P_4_ (−2.28 eV), implying stronger intermediate adsorption capacity. More importantly, this charge redistribution enables the effective integration of electrophilic Co sites (electron-deficient) and nucleophilic P sites (electron-rich) within a single catalyst. The nucleophilic P sites regulate ΔG_H*_ to −0.114 eV, which is close to thermoneutral, thereby promoting water dissociation and the adsorption–desorption equilibrium of hydrogen intermediates. Meanwhile, the electrophilic Co sites optimize the adsorption configuration of the N_2_H_4_ molecule, significantly reducing the free energy barrier of the rate-limiting step of HzOR (*N_2_H_2_ → *N_2_H) from 0.787 eV for pure CoP and 1.06 eV for N-Ni_5_P_4_ to 0.706 eV. When used as a bifunctional electrode for hydrazine-assisted total water splitting, it can drive a current of 10 mA·cm^−2^ at an ultra-low cell voltage of just 0.037 V, with no significant performance degradation observed after 10,000 s of continuous operation. Furthermore, the Ni-Cu-P@Ni-Cu nano-dendritic bifunctional catalyst exhibits a three-dimensional cedar-leaf-like architecture, with a highly porous structure on the electrode surface [73]. This material has a large BET specific surface area and is superhydrophilic (contact angle < 5°), which not only promotes rapid penetration of the electrolyte into the pores to provide sufficient contact area for the reaction but also enables rapid bubble detachment, reducing mass transfer resistance caused by bubble retention. Additionally, the uniform distribution of Ni, Cu, and P within the dendritic structure forms a core–shell architecture consisting of a crystalline Ni-Cu core and an amorphous Ni-Cu-P shell. This configuration ensures efficient electron conduction while providing abundant low-coordination active sites through the amorphous shell. The XPS analysis indicates that the presence of oxidized P species can significantly enhance the electrocatalytic activity of HzOR, thereby proving that the oxidized P species are the active sites. N_2_ bubbles rapidly detach from the surface and diffuse into the surface pores via the electrolyte, resulting in reduced bubble resistance and enhanced charge and mass transfer. This increases the available sites for electrochemical reactions, thereby achieving higher reaction efficiency. In a 1.0 M KOH + 0.5 M N_2_H_4_ electrolyte, a current density of 10 mA·cm^−2^ is achieved at a low potential of only 3.88 mV vs. RHE, and a potential of only 122 mV vs. RHE at 100 mA·cm^−2^. Furthermore, when this catalyst was used as a bifunctional electrode in a hydrazine-assisted hydrogen production electrolyzer, a current density of 10 mA·cm^−2^ was achieved at a cell potential of only 125 mV, far lower than 1.77 V required by conventional total water electrolysis systems, with no significant performance degradation observed after 50 h of continuous operation.

Therefore, by precisely controlling morphology through nanostructure design, it is possible to selectively regulate the morphology and size of materials. Furthermore, these specific morphologies help enhance structural stability, modulate electronic states, promote in situ surface restructuring, and form highly active phases. Through the combined effects of these morphological structures and multiple mechanisms, the rate of catalytic reactions is effectively enhanced.

### 3.2. Metal-Doped Modification

**Figure 3:**
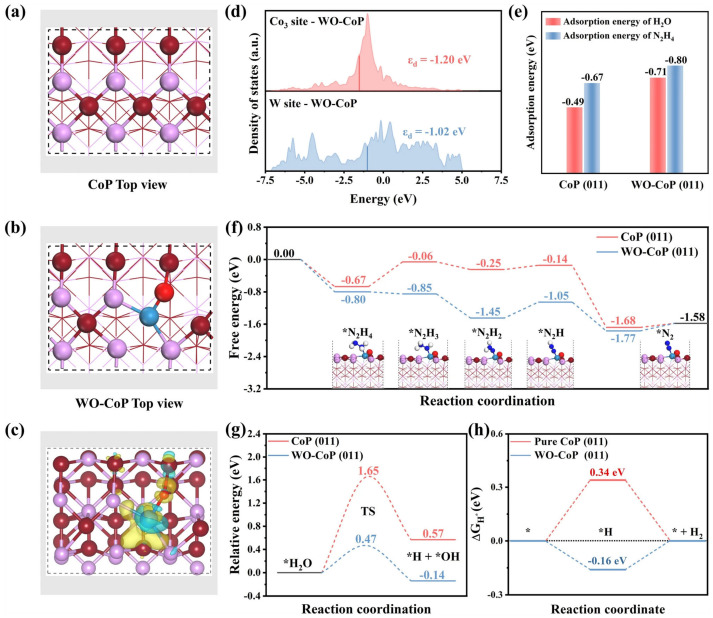
Top views of (**a**) CoP (011) structure model and (**b**) WO–CoP (011) structure model (Co-brownish red, P-pink, W-blue, O-red). (**c**) The calculated charge density difference in WO-CoP. (**d**) Density of states (DOS) of Co_3_ site and W site in WO-CoP. (**e**) Adsorption energy on pure CoP and WO-CoP. (**f**) The free energy diagrams of hydrazine dehydrogenation, (**g**) the free energy diagrams of water dissociation and (**h**) the Gibbs free energy diagrams of pure CoP and WO-CoP. Reprinted from ref. [54].

The catalytic activity of single TMPs catalysts is often limited by their inherent electronic structure characteristics, poor intrinsic conductivity, and a limited number of active sites. By introducing heteroatomic metal atoms to precisely modify the electronic structure of TMPs and optimize the adsorption energy and reaction energy barriers of the catalyst, it is possible to enhance its catalytic performance. Furthermore, doping strategies involving oxygen-containing metal groups can also achieve this modification objective. For example, Meng et al. [54] introduced the highly adsorbent [W–O] group into CoP nanosheets via an in situ hydrolytic etching doping strategy to prepare the 6W–O-CoP/NF bifunctional electrocatalyst (Figure 3a–c) and investigated the performance of this oxygen-containing group in HzOR and HER. As a dedicated adsorption site for H_2_O dissociation and N_2_H_4_ dehydrogenation, the [W–O] group enhances the catalyst’s adsorption of H_2_O and N_2_H_4_. DFT calculations show that [W–O] group doping reduces the energy barrier of the rate-determining step (*N_2_H_2_ → *N_2_H) in HzOR from 0.61 eV to 0.40 eV, and the water dissociation energy barrier in HER from 1.65 eV to 0.47 eV (Figure 3d–h). Concurrently, the 6W–O-CoP/NF catalyst exhibits high electron transfer efficiency, with an electron transfer number of 3.35 for HzOR, approaching the ideal 4-electron transfer, and an electrooxidation efficiency of 85.91%, effectively preventing the formation of harmful NH_3_. This provides an efficient strategy for addressing the generation of harmful gases in HzOR.

**Figure 4:**
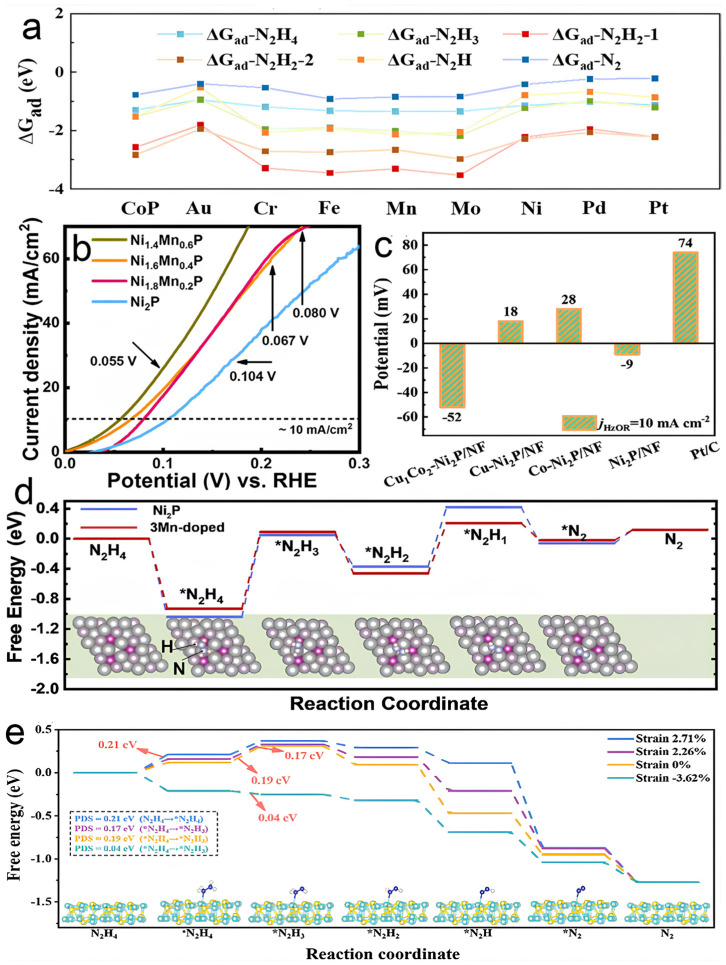
(**a**) The adsorption free energies of CoP–M (Au, Cr, Fe, Mn, Mo, Ni, Pd and Pt) and CoP for six molecules. Reproduced with permission from ref. [35]. Copyright 2025 Royal Society of Chemistry. (**b**) LSV curves of Ni2-xMnxP NCs (x = 0, 0.2, 0.4, and 0.6) for HzOR. Reprinted from ref. [43]. (**c**) Potential of Cu_1_Co_2_-Ni_2_P/NF, Cu-Ni_2_P/NF, Co-Ni_2_P/NF, Ni_2_P/NF and Pt/C in HzOR at a current density of 10 mA·cm^−2^. Reproduced with permission from ref. [74]. Copyright 2023 Wiley. (**d**) The free energy profiles of HzOR for Ni_2_P and 3Mn-doped Ni_2_P and their corresponding stable configurations of the each adsorbed intermediates on the 3Mn-doped Ni_2_P surfaces. Reprinted from ref. [43]. (**e**) Free energy profiles of HzOR on Ni_2_P/NF, Cu_1_Co_2_–Ni_2_P/NF, Co–Ni_2_P/NF, and Cu–Ni_2_P/NF, with 0%, −3.62%, +2.26%, and +2.71% strains, respectively, and the Ni_2_P strain model for adsorption of corresponding intermediates. Reproduced with permission from ref. [74]. Copyright 2023 Wiley.

Metal doping can effectively optimize the electronic structure of TMPs, particularly by modulating the position of the d-band center, thereby precisely regulating the adsorption/desorption behavior of reaction intermediates. Zhou et al. [35] systematically investigated the catalytic regulation mechanisms of CoP doped with eight transition metal single atoms (Au, Cr, Fe, Mn, Mo, Ni, Pd, and Pt) toward HzOR using DFT calculations, identifying Cr and Mn as the optimal dopant elements (Figure 4a). Both elements enhance orbital hybridization between the catalyst and HzOR intermediates by modulating the work function, d-band center, and charge distribution of CoP (Cr-3d hybridizes with Co-3d/P-p, Mn-3d and Co-3d/P-p, respectively), resulting in charge transfer amounts of 0.38 e and 0.31 e upon adsorption of the key intermediate N_2_H_2_^−^, which are higher than the 0.30 e observed for pure CoP, thereby effectively activating the N–H bond. Furthermore, Suryawanshi et al. [43] developed bifunctional Ni_2−x_Mn_x_P NCs by pyrolyzing Mn-doped hollow Ni_2_P nanocrystals. Among these, the optimal Ni_1.4_Mn_0.6_P (x = 0.6) achieved a HzOR current density of 10 mA·cm^−2^ at a working potential of only 55 mV in a 1 M KOH + 0.5 M N_2_H_4_ electrolyte (Figure 4b). The introduction of Mn induces the formation of a hollow nanocrystalline structure via the Kirkendall effect, resulting in a specific surface area of 17.41 m^2^ g^−1^ and an electro-active surface area of 7.91 cm^2^ for Ni_1.4_Mn_0.6_, which is significantly higher than that of pure Ni_2_P. This effectively increases the number of active sites and provides efficient pathways for mass and charge transport. Furthermore, DFT calculations confirm that Mn doping shifts the free energy of hydrogen adsorption (ΔG_H*_) from −0.32 eV in pure Ni_2_P to −0.08 eV, bringing it closer to thermodynamic neutrality. This optimizes the hydrogen adsorption/desorption kinetics of the HER, while simultaneously reducing the reaction energy barrier of the rate-determining step for HzOR (dehydrogenation of N_2_H_4_ to *N_2_H_3_) from 1.09 eV to 1.02 eV (Figure 4d), thereby weakening the binding between the catalyst and N_2_H_4_ and accelerating the dehydrogenation process of hydrazine. Furthermore, this metal doping increases the density of states at the Fermi level of Ni_2_P, enhancing the material’s electrical conductivity and further facilitating charge transfer during the catalytic process.

In terms of bimetallic doping, by regulating the ratio of metal precursors during the synthesis process, it is possible to achieve finer control over the product morphology and pore structure, thereby further enhancing active site exposure and mass transfer efficiency. Feng et al. [74] proposed a simple strategy for engineering the lattice strain of Ni_2_P through Cu/Co dual-cation co-doping, overcoming the limitations of traditional multi-step synthesis methods that induce lattice strain via core–shell structures, and successfully prepared a Cu_1_Co_2_-Ni_2_P electrocatalyst with a compressive strain of −3.62%. The researchers found that single Cu or Co doping induces tensile strains of +2.71% and +2.26% in Ni_2_P, respectively, whereas Cu/Co dual-cation co-doping induces a compressive strain of −3.62% due to strong electron coupling effects. This compressive strain shifts the d-band center of Ni_2_P to −1.79 eV, bringing it closer to the Fermi level. This enhances the adsorption of the catalyst with reaction intermediates, transforming the N_2_H_4_ adsorption process into an exothermic reaction (adsorption energy: −0.21 eV), which offers a thermodynamic advantage over the endothermic adsorption observed in tensile-strained and unstrained Ni_2_P (0.12–0.21 eV) (Figure 4e). Furthermore, this strategy not only lowers the reaction energy barrier for the rate-determining step of HzOR (N_2_H_4_ dehydrogenation to *N_2_H_3_) but also promotes the Volmer step in alkaline HER, bringing the H* adsorption free energy (ΔG_H*_) to −0.09 eV, closer to thermodynamic neutrality, and achieving equilibrium between H* adsorption and desorption, with a current density of 10 mA·cm^−2^ achieved at a low potential of −0.052 V vs. RHE in urea-assisted electrolysis (Figure 4c). This provides new insights for the design of electrocatalysts in related fields.

### 3.3. Heterogeneous Structure Construction

**Figure 5:**
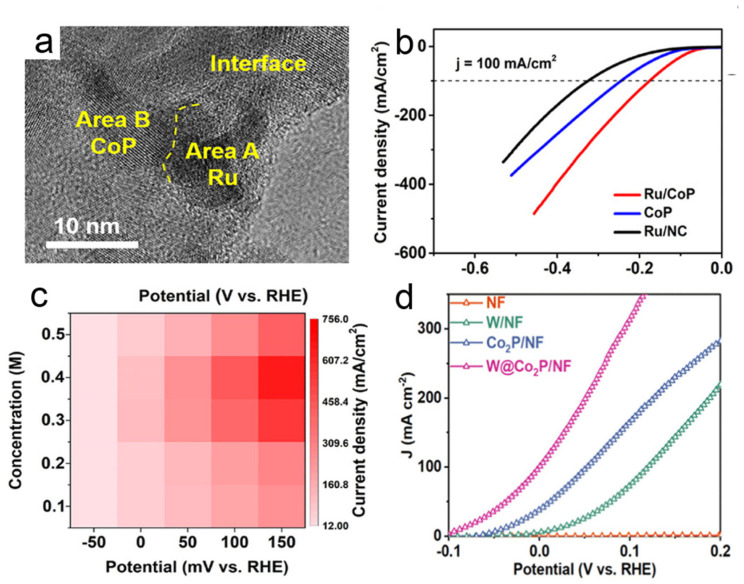
(**a**) HRTEM image of Ru/CoP. (**b**) LSV curves of Ru/CoP, CoP, and Ru/NC for HER. (**c**) Variation in current density with different concentrations of N_2_H_4_ in 1 M KOH for Ru/CoP. Reproduced with permission from ref. [78]. Copyright 2025 Wiley. (**d**) LSV curves in different concentrations of N_2_H_4_ for W@Co_2_P/NF. Reproduced with permission from ref. [79]. Copyright 2023 Royal Society of Chemistry.

Exploiting the difference in work functions between the components of a heterostructure to induce interfacial charge redistribution and modulate the adsorption and desorption of key intermediates is a highly effective strategy [75]. The composition influences the properties of the heterostructure, and the synergy between the two metal phosphides, along with the heterojunction interface they form, optimizes the kinetics of different reaction steps, thereby enhancing catalytic performance. For example, Yan et al. [76] prepared a hetero-structured CoP/MoP catalyst coated with heteroatom-doped carbon (CoP/MoP@NPC) using an organic phosphonate-derived method. XPS analysis shows that electrons flow from CoP (which has a higher Fermi level) to MoP, forming a stable built-in electric field. This charge redistribution not only enhances the overall conductivity but also shifts the d-band center of the CoP/MoP system to a favorable position, thereby lowering the energy barrier for the rate-limiting step (*N_2_H_2_ → *N_2_H) and promoting the dehydrogenation kinetics of hydrazine. At the same time, the heteroatom-doped carbon layer effectively suppresses the agglomeration of phosphide nanoparticles, improving their dispersion and stability. Concurrently, MoP provides a large number of active sites for HER. The P atoms on its surface act as proton acceptors, facilitating the adsorption, activation, and dissociation of water molecules, while the negatively charged P atoms in CoP weaken the metal–hydrogen chemical bonds, promoting hydrogen desorption. Consequently, CoP/MoP@NPC achieved a current density of 10 mA·cm^−2^ at a potential of only 184 mV in HER and a current density of 1 A cm^−2^ in HzOR, achieved at an extremely low potential of just 297 mV (Figure 5a). These results indicate that the heterostructures formed between different phosphide compounds provide more active sites, and the synergistic effects between the components promote enhanced catalytic activity [72,77].

The key feature of transition metal phosphide-precious metal heterostructures is the precise regulation of intermediate adsorption energy through electronic interactions at the interface between the precious metal and the transition metal phosphide, thereby enhancing the bifunctionality of HER and HzOR. Such heterostructures typically exhibit high catalytic activity, with particularly outstanding current density performance at low potentials. Ji et al. [78] first electrodeposited flake-like Co(OH)_2_ onto carbon cloth, then prepared the Ru/CoP heterostructure catalyst (Figure 5b) through ion exchange and phosphorization. The electron transfer at the heterojunction, driven by the work function difference between Ru and CoP, reduces the electron density at the Ru sites, thereby weakening the strong adsorption of H on Ru. Simultaneously, the spontaneous hydrogen overflow effect increases the H coverage at the CoP sites, compensating for the insufficient water electrolysis capacity of CoP. This enables Ru/CoP to significantly enhance HER activity compared to CoP and Ru/NC, achieving a minimum required potential of 175 mV at a current density of 100 mA·cm^−2^, outperforming CoP (244 mV) and Ru/NC (324 mV) (Figure 5c). On the other hand, the reduced electron density at the Ru sites significantly enhances the adsorption capacity for N_2_H_4_. The change in open-circuit potential of Ru/CoP after N_2_H_4_ injection is far greater than that of CoP and Ru/NC. Furthermore, in situ Raman spectroscopy confirms that Ru/CoP can rapidly adsorb and dehydrogenate N_2_H_4_, allowing the NH_2_ intermediate to be efficiently consumed, whereas NH_2_ accumulates in pure Ru/NC. DFT analysis further indicates that the Ru-N bond in Ru/CoP has a higher bond strength, making the N–H bond in the *N_2_H_3_ intermediate easier to break. The dehydrogenation process at the rate-limiting step of HzOR proceeds spontaneously on Ru/CoP, much faster than on pure Ru. This enhanced N_2_H_4_ adsorption and improved dehydrogenation kinetics are key to Ru/CoP’s higher HzOR activity compared to its OER activity. At current densities of 50 and 100 mA·cm^−2^, the potential differences were 1.594 V and 1.612 V (Figure 5d), respectively.

In heterostructures formed by transition metal phosphides and non-phosphide transition metals, the non-phosphide metals (such as W and Ni) primarily serve to modulate electron transport and enhance electrical conductivity, while the transition metal phosphides perform the main catalytic functions. Li et al. [79] in situ fabricated a W@Co_2_P/NF heterostructure electrocatalyst on the surface of nickel foam using a simple electrochemical co-deposition process. The interfacial regulation of W optimized the electron density on the Co_2_P surface, making high-valent Co sites the primary active centers for HzOR, while surface P sites reduced the reaction energy barrier for hydrazine oxidation by modulating the adsorption energy of reaction intermediates, enabling the W@Co_2_P/NF electrocatalyst to achieve a potential of only 51 mV at a current density of 150 mA·cm^−2^. Furthermore, Liu et al. [50] reported a heterojunction material (a-Ni_x_P/Ni/NF) composed of amorphous Ni_x_P and crystalline Ni nanoparticles as a high-performance electrocatalyst for alkaline H_2_O_2_ reduction. The heterointerface formed between Ni_x_P and Ni creates multifunctional active sites, where amorphous Ni_x_P is primarily responsible for the adsorption and activation of N_2_H_4_, while the nanocrystalline Ni promotes the involvement of OH^-^, and together they optimize the adsorption-conversion equilibrium of reaction intermediates. These combined properties demonstrate significant potential for transition metal phosphide–non-phosphide transition metal heterojunctions in electrocatalytic applications.

### 3.4. Other Strategies

**Figure 6:**
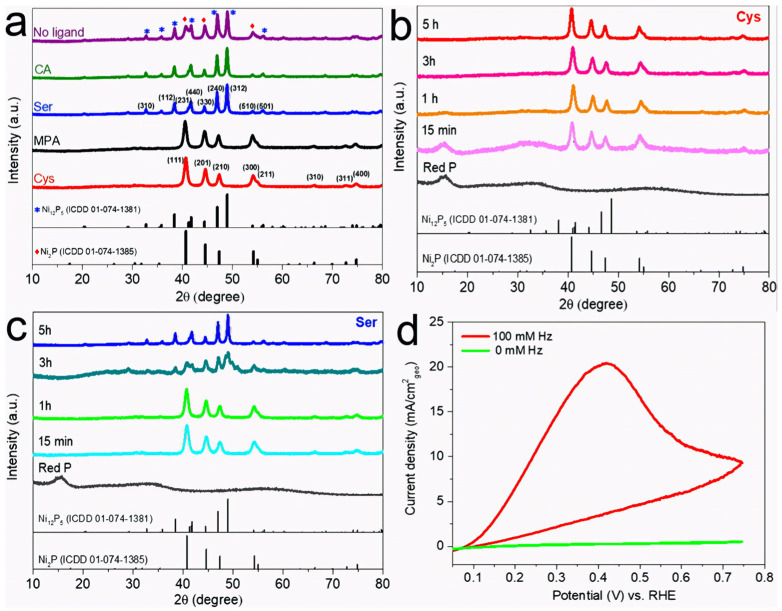
(**a**) Powder X-ray diffraction (PXRD) patterns showing the role of ligands in tuning the phase of nickel phosphides. PXRD patterns of nickel phosphides synthesized at different reaction times using (**b**) serine ligand and (**c**) cysteine ligand reveal the phase transformation process with different ligands. (**d**) LSV curves of Ni_2_P-Cys with Hz and without Hz for HzOR. Reproduced with permission from ref. [80]. Copyright 2022 American Chemical Society.

Local restructuring strategies can precisely control the phase composition on the catalyst surface, thereby effectively activating catalytic active sites in hydrazine-assisted electrolysis systems. At the anode operating potential, TMPs generally undergo dynamic surface reconstruction, with phosphorus selectively leaching out in the form of PO_4_^3–^/HPO_4_^2–^, while transition metal sites undergo hydroxylation and are in situ converted into metal hydroxides (e.g., CoOOH, NiOOH). This dynamic process can be supported by in situ Raman spectroscopy and in situ X-ray absorption fine structure (XAS) measurements. The evolution of Raman characteristic peaks from P–M vibrations to M–O vibrations, as well as the high-energy shift in the absorption edge and the enhancement of the Co–O coordination peak in XAS, all reveal the transformation of the surface phase from phosphides to hydroxidoxides [34,44]. Wei et al. [44] induced local restructuring through chemical reduction to construct the CoH-CoPv@CFP, in which an outer layer of amorphous Co(OH)_2_ coexists with an inner layer of P-vacancy CoP. XPS and XANES/EXAFS characterizations revealed that the valence state of surface Co increased after reconstruction, and the coordination environment shifted from Co–P to Co–O, while the internal CoP framework remained, confirming the formation of P vacancies and the selective enrichment of hydroxyl oxides. This spatially separated structure endows the catalyst with bifunctional properties: the electron-deficient Co sites in the outer layer (resulting from the high electronegativity of O) form stronger N-Co orbital hybridization with N_2_H_4_ molecules, reducing the energy barrier of the rate-determining step of HzOR (N_2_H_4_ → N_2_H_3_) from 0.72 eV to 0.59 eV, while moderately lowering the d-band center, thereby optimizing the intermediate adsorption strength. The inner-layer P vacancies, in turn, adjust ΔG_H*_ from −0.37 eV to a near-thermoneutral value of 0.02 eV, significantly enhancing HER activity. Consequently, at 10 mA·cm^−2^, this catalyst exhibits an HzOR potential of only −61 mV and an HER overpotential of only −77 mV. This strategy effectively addresses the issues of insufficient delocalization of active sites and limited electron transfer distances in traditional electronic regulation, thereby achieving separate optimization of the HzOR and HER pathways. Furthermore, the ligand-tuning strategy enables the rapid and precise selective preparation of nickel phosphide phases, providing low energy consumption, highly stable electrocatalysts for hydrazine-assisted hydrogen production. For example, Praveen et al. [80] introduced thiol (–SH) and carboxylate (–COO^−^) functional groups into the ligands, which effectively modulated the energy levels of the nickel phosphide phases during hydrothermal synthesis. In the absence of ligands, a two-phase mixture was obtained. Thiol ligands (cysteine, 3-mercaptopropionic acid) stabilized the kinetic product Ni_2_P, while carboxylate ligands (citric acid, L-serine) promote the transformation of Ni_2_P into the thermodynamically stable phase Ni_12_P_5_, thereby enabling the selective preparation of pure Ni_2_P and Ni_12_P_5_ phases (Figure 6a–c). Cysteine-modified Ni_2_P exhibits excellent geometric structure and intrinsic electrocatalytic activity under alkaline conditions. In HzOR, a potential of 0.24 V vs. RHE is required at a current density of 10 mA·cm^−2^ (Figure 6d). In commercial applications, the long-term stability of the catalyst is a primary consideration. The introduction of a carbon matrix endows the catalyst with a one-dimensional conductive framework, enhancing electron transport efficiency, while the carbon layer protects the phosphide nanoparticles from electrolyte corrosion, thereby improving catalytic stability. Zhang et al. [77] synthesized the (Co_0.6_Ni_0.4_)_2_P@PC catalyst via a one-step annealing method, in which CoNi bimetallic phosphide nanoparticles are embedded in a phosphorus-doped carbon matrix. After a 10 h long-term test, the current density showed only a slight decrease. Furthermore, the introduction of the carbon matrix ensures uniform dispersion of the bimetallic phosphide nanoparticles, significantly increasing the exposure of active sites, and the formed P–C bonds provide additional sites for the adsorption of reaction substrates and intermediates. XPS results show that the electron transfer from Co/Ni to P causes the metal sites to become positively charged, thereby enhancing their adsorption of N_2_H_4_, while simultaneously causing the P sites to become negatively charged, which promotes the breaking of the N–H bond. These two effects work synergistically to lower the formation energy barrier of the *N_2_H_3_ intermediate. Electrochemical testing indicates that (Co_0.6_Ni_0.4_)_2_P@PC exhibits excellent bifunctional catalytic performance for HER and HzOR in alkaline electrolytes, with a HER overpotential of only 67.9 mV at a current density of 10 mA·cm^−2^ and an HzOR potential as low as −83 mV at a current density of 10 mA·cm^−2^. Additionally, the phosphorus atoms in TMPs can serve as coordination anchors for isolated metal atoms, forming well-defined M-P_4_ units, thereby modulating the d-band center and enhancing the adsorption of key HzOR intermediates. For example, Hu et al. [81] anchored Ru single atoms on NiCoP nanowires, with each Ru atom coordinated to four P atoms and two Ni/Co atoms (Ni(Co)_2_-Ru-P_4_). In this configuration, single-atom phosphorus (the P atoms in the Ru-P_4_ coordination shell) not only firmly anchors the Ru single atom within the phosphide lattice but also induces charge redistribution, lowering the d-band center and enhancing the adsorption of *N_2_H_2_. This reduces the energy barrier of the rate-determining step from 1.28 eV to 0.16 eV. Consequently, Ru_1_-NiCoP requires only −60 mV for the HzOR potential at 10 mA·cm^−2^.

In summary, although doping, heterojunctions, and vacancy engineering differ in their specific mechanisms of action, they all follow the same design principle for selectivity control in addressing the key issue of regulating HzOR selectivity and suppressing N-N bond cleavage to form NH_3_: namely, through atomic-scale localized charge rearrangement and changes in the coordination environment, precisely controlling the central position of the d-band in the active site, thereby altering the adsorption configuration of the N_2_H_4_ molecule and ultimately determining the competitive reaction energy barrier between N-N bond cleavage and N–H bond cleavage. Specifically, on unmodified TMP surfaces (such as pure CoP or Ni_2_P), N_2_H_4_ tends to adsorb in a terminal configuration where a single N atom bonds to the metal site. In this configuration, the electron density of the N–N bond is asymmetrically polarized, resulting in a relatively low activation energy barrier for its cleavage, which facilitates the formation of the NH_2_ intermediate and subsequent hydrogenation to form NH_3_. However, upon the introduction of dopant atoms, the formation of heterojunctions, or the creation of phosphorus vacancies, the localized electron density and geometric structure of the active sites change, prompting N_2_H_4_ to adopt a bridging adsorption configuration in which both N atoms interact with the surface simultaneously. This configuration results in a more uniform charge distribution on the N–N bond, significantly increasing the activation energy barrier required for its cleavage. At the same time, the energy barrier for N–H bond cleavage (particularly the rate-determining step) remains largely unchanged or is slightly reduced. DFT free energy calculations are a key tool for evaluating the aforementioned regulatory effects. On effectively modulated catalyst surfaces, the activation energy barrier for N–N bond cleavage is typically 0.3–0.6 eV higher than that on unmodified surfaces. Meanwhile, the activation energy barrier for the rate-determining step of the ideal 4e^−^ dehydrogenation pathway remains at a relatively low level of 0.4–0.7 eV [35]. The larger the difference between these two energy barriers, the more favorably the reaction proceeds via the N_2_ formation pathway, resulting in lower NH_3_ selectivity.

## 4. HzOR Applications

### 4.1. Direct Hydrazine Fuel Cells

In the energy sector, the most prominent application of the hydrazine oxidation reaction is as the anode reaction in direct hydrazine fuel cells (DHzFC). Compared to traditional hydrogen-oxygen fuel cells, DHzFCs use liquid hydrazine as fuel, thereby avoiding the safety challenges associated with hydrogen storage and transportation, while offering a theoretical cell voltage as high as 1.56 V and an ultra-high theoretical energy density of approximately 4269 Wh/L [82,83].

A DHzFC typically consists of an ORR electrocatalyst at the cathode and an HzOR electrocatalyst at the anode [30].

(10) $$\begin{array}{c} Overall\;reaction:N_{2}H_{4}+O_{2}\to N_{2}+2H_{2}O \end{array}$$

(11) $$\begin{array}{c} Anode\;reaction:N_{2}H_{4}+4OH^{-}\to N_{2}+4H_{2}O+4e^{-} \end{array}$$

(12) $$\begin{array}{c} Cathode\;reaction:O_{2}+2H_{2}O+4e^{-}\to 4{OH}^{-} \end{array}$$

In the DHzFC system, the formation of HzOR at the anode is a key step that determines the overall performance of the battery. An ideal catalyst must possess high intrinsic activity, high selectivity (to avoid the generation of harmful byproducts such as NH_3_), and long-term operational stability. Transition metal phosphides (such as Ni_2_P, CoP, and FeP) exhibit electron properties similar to those of noble metals. Researchers have significantly enhanced the performance of these electrocatalysts by designing nanostructures with high specific surface areas and multiple active sites [53,84]. Sun et al. [85] investigated the application of carbon paper-supported cobalt phosphide nanowire array electrocatalyst (CoP-NWA@CP) in DHzFCs. The catalyst’s 3D nanowire array structure creates a superhydrophobic surface, with an N_2_ bubble contact angle of 150° and bubble adhesion force below the detection limit. This allows bubbles to rapidly desorb within 300 ms while maintaining a diameter of no more than 50 μm, effectively preventing bubbles from covering active sites. Additionally, the 3D array provides a large specific surface area and abundant active sites, accelerating electrolyte diffusion as well as electron and mass transfer. From a practical application perspective, the CoP-NWA@CP catalyst exhibits significant commercial potential in terms of both cost and energy efficiency. This catalyst consists of abundant non-precious elements cobalt and phosphorus, and is fabricated through a facile low-temperature phosphidation route, leading to raw material costs substantially lower than commercial Pt/C catalysts. On the fuel side, hydrazine is a liquid at room temperature and atmospheric pressure. Compared to gaseous hydrogen fuel, it does not require high-pressure storage tanks or liquefaction equipment, which can significantly reduce system infrastructure investment and operating costs. Although hydrazine itself is toxic, its convenience in storage and transportation still gives it a unique competitive advantage. More importantly, in terms of energy efficiency, the DHzFC assembled with this catalyst achieves an open-circuit voltage of 0.905 V, with a peak power density of 300 mW·cm^−2^, whereas a commercial Pt/C-based fuel cell under the same conditions achieved only 61 mW·cm^−2^. Furthermore, after 12.5 h of continuous operation at a current density of 635 mA·cm^−2^, the power density remained at 285 mW·cm^−2^, with a retention rate of up to 95%. Evaluations of the aforementioned performance are primarily based on laboratory-scale membrane electrode assemblies (MEAs), which typically use a cation-exchange membrane (CEM) or an anion-exchange membrane (AEM) as the electrolyte separator However, during actual DHzFC operation, hydrazine molecules (and possibly the intermediate product NH_3_) permeate from the anode to the cathode through the ion-exchange membrane, which not only results in fuel waste and a decrease in open-circuit voltage but also poisons the cathode catalyst (such as Pt/C), severely reducing the fuel cell’s durability [86,87]. Qin [88] et al. systematically compared the permeation behavior of hydrazine in AEM and CEM, confirming that AEM can effectively reduce cross-permeation, although their long-term stability still needs improvement. Furthermore, the compatibility of the copolymer with TMPs catalysts and the optimization of the catalyst layer’s pore structure are crucial for mass transfer at the three-phase interface and bubble evacuation. Therefore, combining TMP anode catalysts with high-performance AEM and a well-designed MEA structure is a key engineering challenge for the practical application of DHzFCs.

**Figure 7:**
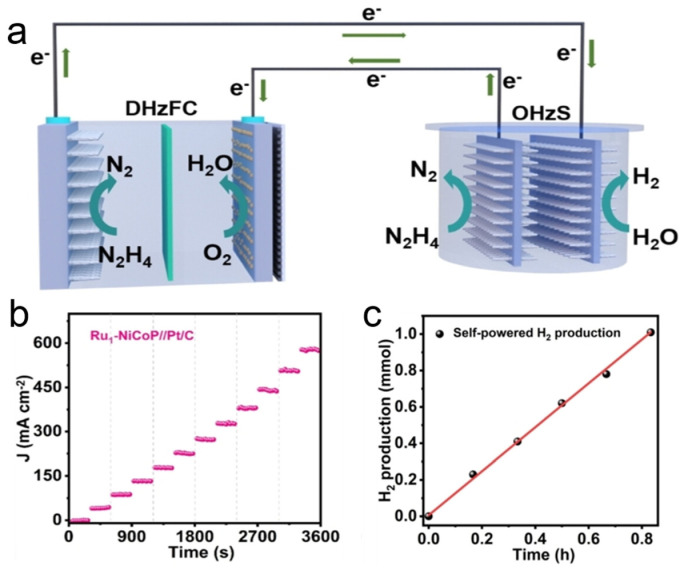
(**a**) Schematic illustration of a self-powered hydrogen production system integrated by driving the OHzS electrolyzer with DHzFC as the power supply. (**b**) Discharge plateaus of Ru_1_-NiCoP at various current densities from 0 to 600 mA cm^−2^. (**c**) Generated amounts of H_2_ from the self-powered H_2_ production system. Reproduced with permission from ref. [81]. Copyright 2023 Wiley.

In addition, a self-powered hydrazine-to-hydrogen generation system fueled solely by hydrazine has recently been proposed. This system utilizes a DHzFC as the power source for an HzOR-assisted hydrogen production system, effectively addressing the issue that traditional HzOR-assisted hydrogen production systems still require an external voltage input, thereby laying the foundation for their application in practical scenarios such as portable devices and vehicles. For example, Hu et al. [81] developed a Ru-modified twisted NiCoP nanowire array (Ru_1_-NiCoP) electrocatalyst that serves simultaneously as the anode for the DHzFC and as the anode-cathode material for the overall hydrazine splitting (OHzS) electrolyzer (Figure 7a), endowing the system with excellent performance. The DHzFC, using Ru_1_-NiCoP as the anode and Pt/C as the cathode, exhibits a high open-circuit voltage of 0.93 V at room temperature and achieves an ultra-high peak power density of 176 mW·cm^−2^ at a cell voltage of 0.40 V. This power density is 3–4 times higher than that of previously reported DHzFCs at room temperature. It exhibits a step-like discharge curve (Figure 7b) across a wide current density range of 0–600 mA·cm^−2^, demonstrating excellent cycling stability. Furthermore, it can operate stably for 4 h at a current density of 40 mA·cm^−2^ with controllable hydrazine fuel consumption. Using this high-performance DHzFC as the power source to drive an OHzS electrolyzer based on Ru_1_-NiCoP, a self-powered hydrogen production system was established. The OHzS electrolyzer itself has demonstrated exceptional hydrogen production performance, achieving a current density of 50 mA·cm^−2^ at an ultra-low cell voltage of just 90 mV, and reaching an ultra-high current density of 522 mA·cm^−2^ at 0.3 V, significantly reducing power consumption compared to conventional water electrolysis systems. Leveraging the high-efficiency power output of the DHzFC, this self-powered system achieves an outstanding hydrogen production rate of 24.0 mol h^−1^ m^−2^ at room temperature (Figure 7c). It is precisely due to the DHzFC’s advantages, including high power density, high open-circuit voltage, and stability across a wide range of current densities, this self-powered system can eliminate dependence on external power sources and enable efficient, stable hydrogen production.

### 4.2. HzOR-Assisted Seawater Electrolysis System

**Figure 8:**
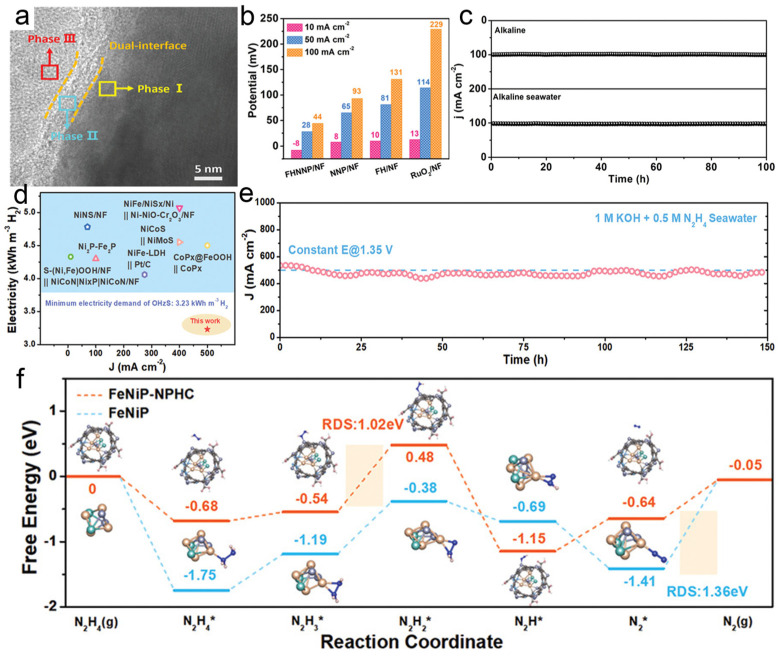
(**a**) HRTEM image of FHNNP/NF. (**b**) Overpotentials at 10, 50, and 100 mA·cm^−2^ of FHNNP/NF, NNP/NF, NNP/NF, and RuO_2_/NF in alkaline seawater for HzOR. (**c**) Chronopotentiometry curves of FHNNP/NF. Reproduced with permission from ref. [92]. Copyright 2023 Wiley. (**d**) A comparison of FeNiP-NPHC (champagne region) with the state-of-the-art seawater/water electrolyzer (pale blue region) in electricity expense and current density. (**e**) The stability test of FeNiP-NPHC toward OHzS. (**f**) The free-energy profiles of HzOR on the FeNiP and FeNiP-NP/C surfaces, the insets are the most stable configurations of the each adsorbed intermediate. Reproduced with permission from ref. [93]. Copyright 2022 Wiley.

Compared to scarce freshwater resources, seawater accounts for 96.5% of the Earth‘s total water supply; its abundance and economic viability may play a key role in driving the industrialization of energy-efficient hydrogen production [89,90]. Consequently, seawater electrolysis technology has attracted widespread attention from numerous researchers. However, under alkaline conditions and at industrial-scale current densities (500–1000 mA·cm^−2^), the chlorine evolution reaction (CIER; 1.71 V vs. RHE) occurring at the anode has a lower kinetic barrier than OER and exhibits easier-to-control reaction selectivity. The competitive relationship between the two hinders the progress of hydrogen production via seawater electrolysis [91]. To address issues such as CIER, HzOR possesses greater thermodynamic advantages than OER, can be employed as a substitute for OER in seawater electrolysis for hydrogen production. On the other hand, using low-cost seawater can address the issue of limited freshwater resources, reduce raw material costs, and further enhance the economic viability of the HzOR-assisted seawater hydrogen production system. Furthermore, various transition metal phosphide-based electrocatalysts with suitable d-band structures have been designed and prepared for the hydrazine oxidation reaction. Most of these catalysts also exhibit activity toward the cathodic hydrogen evolution reaction, thereby demonstrating excellent bifunctionality. Here, Zhang et al. [92] synthesized the foam-nickel-supported FeOOH/Ni_12_P_5_/Ni_2_P three-phase heterogeneous catalyst FHNNP/NF using a two-step method combining low-temperature phosphorization and electrodeposition. Furthermore, the researchers established a dual-BEF (Bias-Induced Electric Field), which is distributed in opposite directions within the FeOOH/Ni_12_P_5_/Ni_2_P ternary structure. One outward-directed BEF originates from the FeOOH/Ni_12_P_5_ interface, while the other inward-directed BEF arises at the Ni_12_P_5_/Ni_2_P interface (Figure 8a). Spectroscopic characterization results confirm that the dual-BEF effect precisely modulates the electronic configuration of the Fe(III) site’s d orbitals, optimizing the binding strength with the N_2_H_4_ intermediate and promoting its activation, thereby enhancing the hydrazine oxidation reaction to achieve energy-efficient hydrogen production. Notably, this significant promotion effect enables the catalyst to exhibit excellent HzOR activity, achieving operating potentials of −8 mV and 44 mV at 10 and 100 mA·cm^−2^ in alkaline seawater, respectively, while maintaining outstanding long-term stability for over 100 h (Figure 8b,c). In a hybrid seawater electrolyzer (HSE) assembled from FHNNP/NF (+) ||NNP/NF (−), cell voltages of 0.22, 0.33, and 0.38 V were achieved at current densities of 10, 50, and 100 mA·cm^−2^, respectively. Under the same conditions, conventional alkaline seawater electrolyzers (ASE) require higher cell voltages of 1.70, 1.81, and 1.86 V, respectively, indicating that HzOR-assisted seawater electrolysis reduces energy consumption.

Furthermore, Yu et al. [93] used a hydrothermal–pyrolysis–phosphidation process to in situ construct a FeP/FeNi_2_P@nitrogen-phosphorus co-doped 1D/3D hierarchical carbon triphase heterojunction electrocatalyst (FeNiP/NPHC) on nickel foam. This catalyst is a bifunctional electrocatalyst for both HzOR and HER and can be used in a self-driven hydrazine-based seawater electrolysis system. At a current density of 100 mA·cm^−2^, the cell voltage of this system is significantly lower than that of conventional total water electrolysis, with a hydrogen production energy consumption as low as 3.23 kWh m^−3^. It can operate stably for 150 h with a Faradaic efficiency approaching 100% (Figure 8d,e). DFT calculations indicate that the heterojunction interface and hierarchical structure effectively modulate the d-band center and electronic structure, optimizing the adsorption energies of N_2_H_4_ and hydrogen intermediates, resulting in a ΔG_H*_ of 0.30 eV for HER that is closer to the thermoneutral value and significantly enhancing catalytic kinetics (Figure 8f). Similarly, Liu et al. [94] employed a two-step hydrothermal and low-temperature phosphorization process to prepare a CoP/Ni_2_P heterojunction electrocatalyst (CoP/Ni_2_P@NF) on foam nickel. The synergistic interaction between CoP and Ni_2_P optimized the electronic structure of active sites, endowing the material with excellent HzOR and HER performance. A two-electrode alkaline seawater electrolysis system for complete hydrazine decomposition based on this catalyst required a cell potential of only 0.108 V at 100 mA·cm^−2^, which is significantly lower than the 1.695 V typically observed in conventional alkaline seawater electrolysis. Furthermore, the system operated stably for 48 h at high current densities ranging from 100 to 500 mA·cm^−2^, providing a design concept for efficient and stable heterostructured catalysts for energy-saving seawater electrolysis-based hydrogen production. When scaling up to industrial-level current densities (>500 mA·cm^−2^), the choice of electrolyzer configuration becomes critical. Traditional H-type two-compartment electrolyzers are not well-suited for practical seawater electrolysis due to their large electrode spacing and high ohmic polarization. In contrast, zero-gap electrolyzers can significantly reduce the ion transport distance by tightly bonding the membrane to the catalytic layer [95]. However, the generation of large amounts of H_2_ and N_2_ bubbles at high current densities increases ohmic resistance and blocks active sites. Therefore, flow plate design (such as serpentine or parallel channels) and the superhydrophobic surface properties of the electrodes are crucial for rapid bubble desorption.

### 4.3. Industrial Wastewater Purification

**Figure 9:**
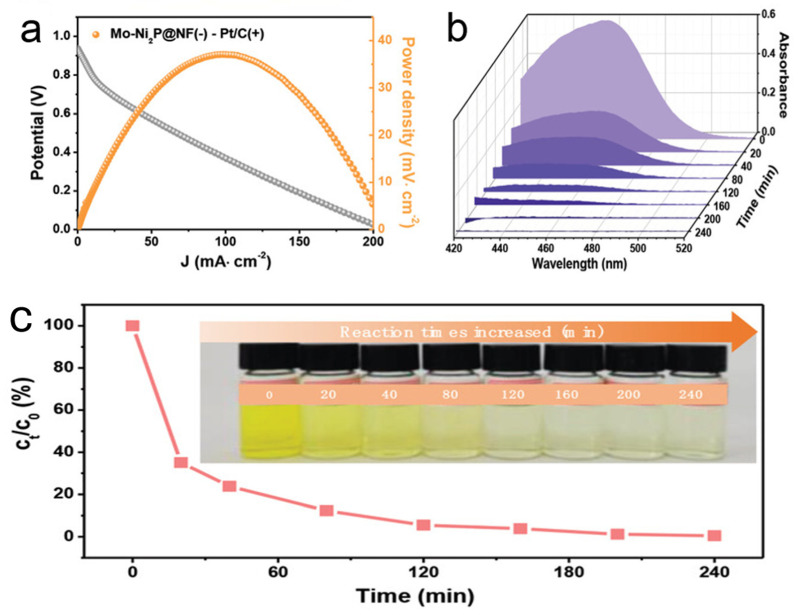
(**a**) Discharge polarization curve and power density plot of DHzFC. (**b**) UV-vis spectra image of the colorimetric industrial N_2_H_4_ wastewater assay of the anolyte after electrolysis for different times at 500 mA·cm^−2^. (**c**) Optical image of the colorimetric industrial N_2_H_4_ wastewater assay of the anolyte after electrolysis for different times at 500 mA·cm^−2^. Reproduced with permission from ref. [98]. Copyright 2023 Wiley.

As a key raw material in industrial sectors such as rocket propellants, pesticides, and chemical synthesis, the production and use of hydrazine inevitably generate large volumes of hydrazine-containing wastewater, with hydrazine concentrations in industrial wastewater typically ranging from 5% to 10%. Conventional methods for treating hydrazine-containing wastewater have significant limitations: physical adsorption methods are prone to causing secondary pollution. Chemical oxidation methods (such as ozone or sodium hypochlorite oxidation) suffer from high oxidant consumption and the toxicity of byproducts (such as nitrosamines). Biological methods are ineffective due to the high toxicity of hydrazine, making them unsuitable for high-concentration wastewater [96]. In recent years, the oxidation of hydrazine has shown application potential in achieving the harmless conversion of hydrazine while reducing energy consumption, as the products are free of carbon contamination and the risk of catalyst poisoning is low [97]. Feng et al. [74] used synthesized Ni_2_P as a catalyst in an HzOR-assisted hydrogen production system. Since HzOR requires only a 0.3% concentration of hydrazine in the electrolyte, industrial wastewater can be used directly for HzOR-assisted hydrogen production. Furthermore, the Mo-Ni_2_P_5_@MNF is applied in a HzOR-assisted hydrogen production system [98]. This catalyst enables the integration of hydrazine-assisted seawater hydrogen production with direct hydrazine fuel cells and solar cells, achieving self-powered hydrogen production with a maximum power density of 37.06 mW·cm^−2^ (Figure 9a). Concurrently, it efficiently treats industrial hydrazine wastewater, rapidly degrading hydrazine concentration to approximately 5 ppb at 500 mA·cm^−2^, which is below the U.S. Environmental Protection Agency‘s 10 ppb limit, with a Faradaic efficiency approaching 100% (Figure 9b,c). Furthermore, Yang et al. [99] constructed a Ni(OH)_2_/Ni_2_P/NF heterogeneous catalyst through interface engineering, achieving an HER potential of −72 mV and an HzOR potential as low as −14 mV at 10 mA·cm^−2^. The cell potentials for hydrazine decomposition at 100 mA·cm^−2^ and 200 mA·cm^−2^ were 0.357 V and 0.513 V, compared to 1.59 V required by conventional water electrolysis at the same current density (100 mA·cm^−2^), representing a significant energy savings. The heterogeneous interface of the catalyst induces charge redistribution, thereby optimizing the adsorption energy of intermediates. Concurrently, the multi-level porous structure significantly increases the active surface area, with an apparent activation energy as low as 30.7 kJ·mol^−1^. These characteristics enable the catalyst to be adapted to a batch-type hydrazine wastewater treatment mode, capable of degrading industrial hydrazine wastewater concentrations to below 0.03 M, simultaneously achieving energy-saving hydrogen production and wastewater purification without the need for additional oxidants. The above transition metal phosphide catalyst, composed of elements abundant in the Earth’s crust, can self-grow on a foam nickel substrate without the need for precious metals such as Pt or RuO_2_ or additional binders, thereby effectively reducing electrode manufacturing costs. Consequently, compared to conventional water electrolysis for hydrogen production, this water electrolysis system coupled with HzOR not only offers significant economic benefits but also simultaneously provides environmental benefits through the purification of hydrazine wastewater.

## 5. Future Prospects and Challenges Ahead

Although TMP-based catalysts have achieved remarkable results in this field, numerous challenges remain in scaling them from laboratory to industrial applications. Future research should focus on the following directions:

Firstly, current mainstream synthesis strategies (e.g., liquid-phase methods, gas–solid reactions) often rely on toxic, expensive phosphorus sources (e.g., TOP, NaH_2_PO_2_) and harsh reaction conditions, whose safety and environmental costs constrain large-scale production. Developing green, low-toxicity phosphorus sources and precursors, alongside exploring mild, energy-efficient preparation pathways like plasma-assisted or photocatalytic synthesis, is key to achieving sustainable TMPs production. Secondly, the understanding of the true active site structure of TMPs under actual operating conditions, their dynamic evolution during reactions (e.g., surface reconstruction, phase transitions), and interfacial charge transfer mechanisms remains incomplete. Future efforts should integrate in situ/operational characterization techniques (e.g., XAS, Raman, APXPS) with theoretical calculations to reveal the intrinsic structure-activity-stability relationships at the atomic/electronic level, guiding rational design of high-performance catalysts. Thirdly, most current studies are conducted under ideal laboratory conditions, such as high hydrazine concentrations and high-purity electrolytes, which differ significantly from actual industrial scenarios characterized by hydrazine concentrations as low as the ppm level, complex impurity interference, and high current density operation. Future research should establish testing standards that closely mimic industrial conditions, develop TMP catalyst systems capable of maintaining high activity at ppm-level hydrazine concentrations, and systematically evaluate the impact of impurity ions on catalyst selectivity and stability to advance the practical engineering application of this technology. Fourthly, the hydrazine system itself presents several unique and critical challenges that require targeted solutions. (1) The cleavage of the N–N bond during the hydrazine oxidation reaction may generate NH_3_ as a byproduct, which not only reduces the anode’s Faradaic efficiency but also poisons the cathode HER catalyst. Therefore, catalyst design must balance high selectivity for the four-electron transfer pathway while suppressing the N–N cleavage pathway through component regulation and surface modification. (2) The N_2_H_x_ and NH_x_ intermediates generated during the reaction tend to adsorb strongly onto active sites, leading to gradual catalyst surface poisoning and deactivation. The extent of poisoning is closely related to the potential, hydrazine concentration, and dynamic evolution of the surface structure. This necessitates future research utilizing in situ/operational characterization techniques to thoroughly analyze the adsorption–desorption behavior of nitrogen-containing species and the mechanisms by which they passivate active sites. (3) At the device level, hydrazine and any NH_3_ that may be generated can permeate from the anode to the cathode through the proton exchange membrane, causing a drop in open-circuit voltage and a reduction in Faradaic efficiency. However, current research on membrane material selection (e.g., AEM versus PEM) and strategies to suppress cross-permeation in TMPs-based hydrazine-assisted electrolysis systems remains very limited. Systematic evaluations at the membrane–electrode assembly (MEA) level are needed in the future.

## Data Availability

No new data were created or analyzed in this study. Data sharing is not applicable to this article.
